# Factors Influencing Engagement and Behavioral Determinants of Infant Feeding in an mHealth Program: Qualitative Evaluation of the Growing Healthy Program

**DOI:** 10.2196/mhealth.8515

**Published:** 2017-12-18

**Authors:** Eloise-Kate Litterbach, Catherine G Russell, Sarah Taki, Elizabeth Denney-Wilson, Karen J Campbell, Rachel A Laws

**Affiliations:** ^1^ Institute for Physical Activity and Nutrition School of Exercise and Nutrition Sciences Deakin University Geelong Australia; ^2^ Centre for Obesity Management and Prevention Research Excellence in Primary Health Care Sydney Australia; ^3^ Centre for Advanced Sensory Science, School of Exercise and Nutrition Sciences Faculty of Health Deakin University Geelong Australia; ^4^ Faculty of Health University of Technology Sydney Sydney Australia; ^5^ Health Promotion Unit Sydney Local Health District Sydney, NSW Australia; ^6^ School of Nursing University of Sydney Sydney Australia; ^7^ Sydney Local Health District Sydney Australia

**Keywords:** mobile health, parents, personal satisfaction, behavior, infant, obesity, prevention and control

## Abstract

**Background:**

Infant feeding practices, including breastfeeding and optimal formula feeding practices, can play a role in the prevention of childhood obesity. The ubiquity of smartphone ownership among women of childbearing age provides important opportunities for the delivery of low-cost, broad reach parenting interventions delivered by mobile phone (mHealth or mobile health interventions). Little is known about how parents engage with mHealth programs targeting infant feeding and how such programs might influence infant feeding practices.

**Objective:**

The objectives of this study were to explore participant views on (1) factors influencing engagement with the Growing healthy program, an mHealth program targeting healthy infant feeding practices from birth to 9 months of age, and (2) the ways in which the program influenced behavioral determinants of capability, opportunity, and motivation for breastfeeding and optimal formula feeding behaviors.

**Methods:**

Semistructured, telephone interviews were conducted with a purposeful sample (n=24) of mothers participating in the Growing healthy program. Interviews explored participants’ views about engagement with the program and its features, and the ways the program influenced determinants of infant feeding behaviors related to breastfeeding and optimal formula feeding. The interview schedule was informed by the Capability, Opportunity, Motivation, and Behavior (COM-B) model.

**Results:**

Participants reported that engagement fluctuated depending on need and the degree to which the program was perceived to fit with existing parenting beliefs and values. Participants identified that the credibility of the program source, the user friendly interface, and tailoring of content and push notifications to baby’s age and key transition points promoted engagement, whereas technical glitches were reported to reduce engagement. Participants discussed that the program increased confidence in feeding decisions. For breastfeeding mothers, this was achieved by helping them to overcome doubts about breast milk supply, whereas mothers using formula reported feeling more confident to feed to hunger and satiety cues rather than encouraging infants to finish the bottle. Participants discussed that the program provided around-the-clock, readily accessible, nonjudgmental information and support on infant feeding and helped to reinforce information received by health professionals or encouraged them to seek additional help if needed. Participants reflected that their plans for feeding were typically made before joining the program, limiting the potential for the program to influence this aspect of motivation. Rather, the program provided emotional reassurance to continue with current feeding plans.

**Conclusions:**

Our findings suggest that engagement with the program was influenced by an interplay between the program features and needs of the user. Participants reported that the program enhanced confidence in feeding decisions by providing a 24/7 accessible, expert, nonjudgmental support for infant feeding that complemented health professional advice. It is likely that interventions need to commence during pregnancy to maximize the impact on breastfeeding intentions and plans.

## Introduction

### Child Obesity Prevention and Infant Feeding

Childhood overweight and obesity remains a substantial public health challenge in Australia and internationally, with important health and economic consequences [1]. Children are becoming overweight at a young age, with 22.8% of children aged 2 to 4 years *already overweight or obese* [2]. Infants who grow rapidly during infancy are at increased risk of subsequent obesity in both childhood and adulthood [3,4]. Infant feeding practices, including whether an infant is breastfed (and for how long) [5] and how formula is used (including the protein content of the formula, how much is offered, how it is prepared, feeding on a schedule, and putting infants to bed with a bottle), are all associated with rapid weight gain in infancy [5-9]. Australian data from a 2010 national survey indicated that approximately 10% of Australian infants were exclusively formula fed from birth, 40% had at least some formula by 1 month of age, and only 15% were exclusively breastfed until 6 months of age [10]. Similar proportions are reported in the United States where national rates of exclusive breastfeeding at 6 months are 22% [11]. This clearly highlights the need for interventions to promote longer breastfeeding duration and exclusivity, but given the high rates of formula use, strategies are also required to promote optimal formula feeding practices to prevent rapid weight gain and early onset of obesity.

### mHealth and Infant Feeding

Mobile health (mHealth) interventions present an appealing new avenue to support parents with infant feeding. Smartphone ownership is increasing worldwide [12], with Australia having the highest rate (93%) of access to smartphones [12]. Furthermore, women of childbearing age (18-49 years) spend, on average, around 21 hours a week on their smartphone [13]. Well-designed smartphone apps can provide “around-the-clock” high-quality information as well as personalized and tailored support at low cost and are easily scalable to maximize reach [14]. A key gap identified in our previous qualitative work with mothers [15] was the lack of reliable and practical advice at the exact time of need (eg, breastfeeding support in the middle of the night), highlighting the value of mHealth approaches in the context of infant feeding. Although studies [16-20] suggest that the majority of mothers (ranging from 51 to 97% across studies) use the Internet for information on infant feeding and care, less information is available on the use of apps in the postpartum period. A recent study [21] among low-income women reported that apps were commonly used during pregnancy but not in the postpartum period because of limited availability of high-quality apps, creating a postpartum app gap. In line with this, our own research [22] found that 78% of apps on infant feeding available in Australia were of poor quality because of deficits in navigability, design, readability, breadth of coverage, and author credibility.

### Efficacy and User Engagement With mHealth Interventions

Early research on the efficacy of mHealth interventions in changing health behavior is promising [23-25]; however, there is a paucity of research on the efficacy of such interventions in influencing infant feeding behaviors. A recently published review of mHealth interventions found that only 6 of 23 studies used behavior change theory to inform the development of the app [26]. Given that it is well accepted that interventions underpinned by behavior change theory are more likely to be effective [27-29], this represents an important gap in the mHealth literature.

The same review [26] reported that some features improved the effectiveness of health-related apps. These included if apps were time efficient, easy to use, provided real-time feedback, were individualized, provided detailed information, and included health professional involvement [26]. This suggests that factors influencing user engagement can have a direct bearing on how effective mHealth interventions will be [26]. Engagement is influenced by the attributes of the user, the system, and user-system interaction [30]. Specifically, in mHealth interventions, the mode of delivery (eg, use of push notification and games), content (eg, behavioral targets and use of behavior change techniques), and quality (such as credibility, functionality, aesthetics, and subjective experience) have been shown to influence engagement [31]. Evidence also suggests that interventions designed to address the unique preferences of the participants will have a greater impact on program engagement and subsequent outcomes [32]. The mHealth design and delivery characteristics important in the parent infant feeding domain are poorly understood.

### The Growing Healthy Program

We have recently developed the Growing healthy program, an mHealth intervention for parents of young infants, which encourages healthy infant feeding practices across the first 9 months of life, with a focus on socioeconomically disadvantaged parents. Details about the program and its development have been published elsewhere [33]. Briefly, the program consisted of an app and website, providing parents with a “one-stop shop” for evidence-based advice and strategies that are consistent with national guidelines on infant feeding in the 9 months after birth. The features of the mode of delivery included information (videos, written content, and links), automated messages (3 personalized push notifications or short message service text messages per week, tailored to the infants’ age and feeding mode: breast, formula, or mixed feeding, and a weekly email summarizing the messages), and communication functions (Facebook, sharing content with others). Personalized messages direct users to tailored information (eg, breastfeeding mothers were directed to breastfeeding content), but participants were not restricted from accessing other information (eg, on formula feeding).

The development of the program was guided by the Behavior Change Wheel framework, a well-recognized approach to developing behavioral interventions that takes into account the context in which behaviors occur [34]. To understand infant feeding behaviors, extensive formative work including 2 systematic reviews [35,36] and qualitative interviews with both health practitioners [37] and socioeconomically disadvantaged parents [15] were used to identify the selection of the target behaviors, key determinants of these behaviors in context, and appropriate intervention delivery mode. Determinants of infant feeding behaviors were explored within the domains of capability (eg, skills, knowledge, and confidence), opportunity (eg, access to information or equipment or social and cultural norms), and motivation (eg, habits, emotions, plans, or goals) as outlined in the Capability, Opportunity, Motivation, and Behavior (COM-B) model [34]. Behavior change techniques were mapped to the determinants underlying each behavior using Michie’s taxonomy [38] and were selected if they were feasible to be used in the mHealth format. The design of the app was also informed by best practice principles in mobile health app design [39] with the purpose of addressing key gaps in existing infant feeding apps.

### Study Aims

This qualitative study aimed to explore participant views on (1) factors influencing engagement with the Growing healthy program and (2) the ways in which the program influenced behavioral determinants of capability, opportunity, and motivation for breastfeeding and optimal formula feeding behaviors. The findings from this study will provide important new insights to guide the development of future mHealth interventions targeting infant feeding to maximize behavior change and effectiveness.

## Methods

### The Growing Healthy Feasibility Study

A feasibility study of the Growing healthy program has been conducted to examine the acceptability and preliminary effectiveness using a quasi-experimental design, with an mHealth intervention group and a concurrent nonrandomized comparison group [33]. Participants were recruited to the Growing healthy program in the following 3 ways: via their primary care providers in socioeconomically disadvantaged communities in 2 Australian states, face-to-face by researchers, and through advertising on the Web [40]. Eligibility criteria for participation in the program included the following: pregnant (30+ weeks’ gestation) or parent/main carer of an infant aged under 3 months, snartphone ownership, English literacy, aged 18 years or older, and resident in Australia. Further details of the recruitment process and outcomes have been described elsewhere [40].

### Study Participants

For this qualitative substudy, Growing healthy participants (n=301) were purposefully selected from those expressing interest in participating in an interview about their experiences of using the program when their infants were between 6 and 9 months old. From those who expressed interest (n=67), participants were purposefully sampled to recruit mothers with a range of feeding modes, including breastfeeding, formula feeding, and mixed feeding (combining both breastfeeding and formula feeding) and those who were university or nonuniversity educated. Purposefully selected individuals (n=39) were invited by email to participate. Nonresponders were sent a reminder email 1 week following the initial invitation, and if they were unresponsive, participants were called 1 week later to confirm their interest and to schedule an interview. A total of 24 individuals agreed to take part, the remaining 15 were uncontactable. Data saturation was reached (as determined by no new information emerging) after conducting interviews with all who agreed to participate. Verbal consent to participate was given at the initiation of the interview and a Aus $30 supermarket voucher was provided as compensation for the time taken to complete the interview.

### Data Collection

The interview schedule consisted of semistructured questions tailored to mothers’ feeding mode and the mode of delivery of the Growing healthy program (ie, whether the participant was an app- or website user, used push notifications or text messages, and read the Growing healthy emails). The questions were structured to address the 2 aims of the study (Table 1). First, questions were asked about their engagement with the program and its features. The second part of the interview sought to explore in what ways the program influenced behavioral determinants of capability, opportunity, and motivation for breastfeeding and optimal formula feeding behaviors. Interviews were conducted by 1 author (EL) until saturation was reached (ie, until no new information emerged). Interviews were recorded with participants’ permission and transcribed verbatim by a professional transcription service.

### Data Analysis

Transcriptions were de-identified and cross-checked with the audio file for accuracy. Thematic analysis was performed using the methods of Braun and Clarke [41]. This method starts with the familiarization of the data by reading the interviews, generating initial codes based on the data. In this study, coding was also informed by factors known to influence engagement with mHealth, including mode of delivery, quality, and content as well as the COM-B model for behavioral determinants [34].

**Table 1:** Outline of interview questions.

Domain	Examples of interview questions/prompts
Engagement	*To start with can you tell me if you used:*
	The app? If YES, did you receive any push notifications or messages from the app? Did you read any of these? Did you click on any of the push notifications? How did you mainly use the app?
	The website? Did you receive weekly email with links to the website? Did you ever click on these links?
	*I wonder if you could tell me a little bit about your experience of using the Growing healthy app/website?*
	What did you think of the app/website?
	When did you use the app/website?
	*Let’s look at the home page of the app/website together (as a memory prompt for the questions below)*
	Were there any particular sections that you looked at more than others? Why was that?
	What have been the most helpful sections of the app? Can you tell me about a time when you used it and how it helped?
	Were there any sections of the app which you didn’t find helpful? Can you tell me more about that?
Behavioral determinants	*I would now like to ask you about formula feeding/breastfeeding/mixed feeding and the formula feeding/breastfeeding/mixed feeding section of the app (get them to open it if possible)*
	How helpful was this section of the app/website? Prompts: what did you like? Dislike?
Behavior	In what ways (if any) do you think it changed how you fed your baby?
Capability	What new things (if any) did you learn from the app/website about formula feeding/breastfeeding/mixed feeding?
Motivation	Did it change the way you felt about formula feeding/breastfeeding/mixed feeding?
Opportunity	How well supported overall did you feel in breastfeeding/formula feeding/mixed feeding your baby? To what extent (if any) did the Growing healthy program (app/website/push notification) influence how well supported you felt in breastfeeding/formula feeding/mixed feeding?
Opportunity	Did you seek any additional advice or information on formula/breastfeeding/mixed feeding outside of that received on the app/website? What prompted you to seek this advice or information? How did this section of the app (website or the notifications you received) fit with the advice or information you received from elsewhere? How did you deal with any conflicting advice?
	Do you have any further comments or anything to add about the Growing healthy program and your experience of feeding your baby?

An initial coding manual was devised based on a review of 5 interviews and subsequently revised several times during the coding process, adding new codes as needed until no new codes were identified. Three researchers (EL, CGR, and RL) were involved in developing the coding manual based on reading transcripts individually and meeting to discuss the manual. Upon finalizing the coding manual, all interviews were coded by EL with a subset coded by RL. Minor inconsistencies were identified and were resolved through discussion. The researchers then looked for key themes within the data, and upon reviewing these themes, condensed them where appropriate. Finally, themes were defined and appropriately named. Coding, storing, and sorting of de-identified transcripts was undertaken using QSR NVivo software version 11.

### Ethics and Study Approvals

Ethics approval was provided by Deakin University 2014-093 and University of Technology Sydney 2014000123 *.*

## Results

### Participants

Participant characteristics are shown in Table 2. Of the 24 participants, 13 were breastfeeding, 9 were formula feeding, and 2 were mixed feeding. Half of the sample were university educated (which is representative of the total sample of participants for the feasibility study) and the infants were aged between 25 and 36 weeks at the time of interview. There was no significant difference in sociodemographic characteristics between those who agreed to participate and those who were uncontactable (Table 2). Interviews were conducted over 6 weeks from January to March 2016. The mean duration of the interview was 17 min (range: 13-35 min).

### Participant Views on Factors Influencing Engagement With the Program

Participants in this study reported high engagement with the Growing healthy program. Participants used the program to browse content, to actively search for a particular topic to address an immediate need, or were prompted to use the program from a push notification or text message or email. Most participants indicated that they used the app more than the website because it was more conveniently accessed on their smartphone. Engagement with the program was influenced by a range of factors, including user needs and program features (Table 3).

**Table 2:** Participant and infant characteristics.

Characteristics			n=24
**Participant characteristics**			
	Mean age in years (range)		31 (24-38)
	**Education**		
		University	12
		Non-university	12
	**Country of birth**		
		Australia	22
		United States	1
		Indonesia	1
**Infant characteristics**			
	Mean age in weeks (range)		31 (25-36)
	**Gender**		
		Male	15
	**Feeding methods (in addition to solids)**		
		Breastfeeding	13
		Formula feeding	9
		Mixed feeding (formula and breastfeeding)	2

**Table 3:** Factors influencing engagement with the program: themes and illustrative quotes.

Theme		Illustrative quotes
**User needs**		
	Baby age and transition points	*Yeah at the beginning I didn’t know what I was doing*, *but now I think I’ve got the hang of it. [Participant #23]*
	First-time mother	...*well as a first time mum, I didn’t have any clue because I’ve never been around babies and I’m like “Is this normal?” [Participant #5, first-time parent]*
		*I guess I felt I knew it all by now, with my third [baby] but yeah. Would have gotten a lot of tips if it were my first baby. [Participant #24, 3 children]*
	Vulnerable parents	...*it just really helped me because not really knowing what to do or where to turn to and things like that. It helped me to go on. Like for example the talk to other mums (section) was like “Okay, I’ll join in with my local maternal child health nurse mums group” and make sure I keep going to that. [Participant #6]*
	Congruence with parenting philosophy and beliefs	...*I found it quite good because it goes along with the guidelines of the WHO like the World Health Organization which is what I go by personally. So yeah, I quite liked it*. *[Participant #14]*
		*I guess it’s less that I wasn’t interested and more that I found a lot of the topics didn’t really mesh well with my sort of parenting style or my parenting philosophy kind of thing*. *[Participant #6]*
**Program features**		
	User friendly and easy to navigate	*I think it’s really well organized. Like it’s really user friendly with all the topics and you can go in and it’s really easy to find what you’re looking for... [Participant #10]*
	Credible facilitator	*I found that reading the Growing healthy app I had the confidence that the information was Australian and that it was best practice and that it was put out by a university that has—a couple of universities that have some kind of I guess credence and reliability. So I know that what I was reading wasn’t necessarily just the opinion of some whacko. [Participant #8]*
	Push notifications, email, and text messaging prompt	*It’s like they (the push notifications) were reading my mind, quite often they popped up at the right time when I was actually thinking I wonder what’s going on with X,Y or Z, and that’s when it usually pops up. [Participant #11]*
		*I turned off push notifications for a lot of my apps because I was just getting so many. [Participant #17]*
	Technical issues	*One of the things that did deter me a bit was that the app would just randomly close down. [Participant #9]*

#### User Needs

Engagement with the program fluctuated depending on the mothers’ needs (eg, when she was in need of more support on a particular topic) and on their infant’s stage of development. In particular, participants reported that their engagement was highest when their baby was very young and they were establishing routines (eg, breastfeeding, sleeping) and during times of transition (eg, introducing a bottle, formula, or solids and going back to work). First-time mothers reported using the program as a learning tool, whereas mothers with older children discussed using the program less frequently, typically to reinforce what they had learned with their older child or children. A number of vulnerable parents (those with postnatal anxiety, depression, feeding problems, or those who reported finding the transition to motherhood difficult) reported referring to the app for tips, resources, and reassurance. Participants reported that for their engagement with the program to be high, the content needed to be consistent with their own parenting beliefs and values. For example, if the content was consistent with their extant beliefs about appropriate ways for infants to sleep and feed, they were more likely to engage with the program. In contrast, if the content did not align with their preexisting parenting beliefs and values, they were less likely to engage with the program.

#### Program Features

Most participants thought the app was clear, contained sufficient information, was user friendly, and was easy to navigate. Many participants perceived the quality of the program to be high because 2 credible universities designed it. This encouraged feelings of trust and confidence in the information, which participants felt was important in promoting greater app use. The receipt of push notifications, text messages (for Web users), and emails was important in prompting engagement for some participants, particularly when the messages aligned with participants’ experiences and needs. Others reported difficulty with knowing how to retrieve push notifications (even if they were perceived to be relevant) or switching off push notifications because of the large number received from multiple apps. Other technical glitches, including the failure of the app to work at times, were reported to reduce engagement.

### Participant Views on How the Program Influenced Capability, Opportunity, and Motivation

The key themes arising from participant interviews on how the Growing healthy program influenced capability, opportunity, and motivation for breastfeeding and optimal formula feeding practices are outlined in Table 4 and described below.

#### Capability

Many mothers interviewed reported that the Growing healthy program increased their confidence in feeding decisions. Confidence was increased by the reassurance provided by the program that mothers were engaging in feeding behaviors that were healthy for their infant and they were doing the “right thing.” This was evident for the majority of participants interviewed, regardless of their feeding mode (breast, mixed, and formula). Breastfeeding mothers reported that the program helped confirm they were breastfeeding their baby correctly. These mothers also noted that the program provided them with the confidence to continue breastfeeding, particularly when they doubted their milk supply. Formula and mixed feeding mothers discussed that the app increased their confidence to demand feed following their infants’ hunger and satiety cues rather than encouraging infants to finish the bottle. Confidence was also increased because of the credibility of the information source coming from university experts.

#### Opportunity

Participants discussed that the program provided access to understandable, credible information while also providing social support. Participants particularly commented upon the value of the support provided by the app at times of need such as when they were questioning their milk supply and during times when it was not possible to seek advice from others (eg, in the middle of the night). Participants who were formula feeding or mixed feeding also indicated that the program provided support without fear of judgment of their decision to use formula. These women reported feeling reluctant to discuss formula use with health professionals because of fear of being judged. Some participants noted that the information in the program reinforced advice provided by others in their social and health networks (eg, Midwives, Maternal and Child Health Nurses, and General Practitioners) particularly with regard to breastfeeding. Mothers who were exclusively breastfeeding at the time of the interview were more likely than formula or mixed feeders in this sample to talk about having sought additional help for infant feeding from a range of sources. That is, the program encouraged them to seek additional help if needed, thus potentially increasing both the advice and support they received (opportunity) as well as their skills, knowledge, and confidence in breastfeeding (capability).

#### Motivation

Motivation in the form of plans was rarely mentioned as having been influenced by the Growing healthy program. For example, mothers appeared to have set plans for if and how long an infant would be breastfed and desires to introduce formula, and these were reportedly formed before joining the program. Nonetheless, mothers reported that the program influenced their motivation to continue with their current behaviors by providing reassurance that they were doing the “right thing” for their baby, both nutritionally and for nonfeeding-related behaviors, such as sleeping.

**Table 4:** Participant views on how the Growing healthy program influenced behavioral determinants (ie, capability, opportunity, and motivation): themes and illustrative quotes.

Theme		Illustrative quotes
**Capability**		
	Reassurance—doing the “right thing”	*So the app said about demand feeding and letting them stop when they’re full, etc, which I found really useful because of course when you’re looking in the bottle and they’re not drinking it all you start thinking no, why aren’t they drinking. [Participant #20, mixed feeding mother]*
	Confidence to keep going	*I guess it gave me the confidence to continue even when I was struggling, having issues and starting to doubt myself and doubt that I had enough milk supply. I guess it was just the information that I needed to keep me going*. *[Participant #11, breastfeeding mother]*
	Credibility of provider-enhanced confidence	*I think it gave me more confidence in the decisions I made because I felt like the decisions I made were supported by good information and a reputable distributor of information. [Participant #8, formula feeding mother]*
**Opportunity**		
	24/7 access to clear, credible information and support	...*you could go into that app any hour of the day even if it’s 3 am in the morning and you’re breastfeeding and you want to check something and you got your phone there but none of those primary support people are around because they’re asleep*. *[Participant #17, breastfeeding mother]*
	Support without fear of judgment	*I would never go to my nurse or like any of those things to tell that I was going to stop breastfeeding or anything like that because you tend to get a lecture but it’s nice to have I suppose an information source that’s not very anti formula for a change. That’s yeah—most sources are anti formula. [Participant #4, mixed feeding mother]*
	Reinforced information from other health and social networks	*It was just good information; clear, concise. Similar to some of the other material that I’d been referred to by my maternal health nurse...It was good that at least they were both consistent in the information that they were presenting. [Participant #15, breastfeeding mother]*.
	Encouraged seeking of health professional support	...*reading the app actually directed me to the Australian Breastfeeding Association and a lactation consultant. So that kind of, the app is the one that I recommend that I see them. [Participant #14, breastfeeding mother]*
**Motivation**		
	Feeding plans/intentions already formed before app use	*I don’t think the Growing healthy app really played any part in it. Like I think I started formula feeding before that [having the App], like in that first week. [Participant #12, formula feeding mother]*
	Motivation to continue with current behaviors by providing reassurance	*I was stressed that she wasn’t getting enough milk and reading through that it said so long as she had so many wet nappies and things like that, and she actually had all of the things, like she was fine and I was just overthinking it and stressing it...so then it actually relieved my anxiety of thinking she wasn’t getting enough milk and I was going to give up breastfeeding. [Participant #11, breastfeeding mother]*

## Discussion

### Principal Findings

To our knowledge, this is the first study to explore participant views on factors influencing engagement with an mHealth intervention targeting infant feeding and the ways in which the program reportedly influenced key behavioral determinants of breastfeeding and formula feeding practices. The findings suggest that engagement is influenced by an interplay between the needs of the users, congruence between the program and existing parenting beliefs, and the program features. Participants reported that the program enhanced confidence in feeding decisions by providing an “around-the-clock,” credible, nonjudgmental support for infant feeding that reinforced and complemented information received from social networks and health professionals. Participants reflected that motivation in terms of feeding plans and intentions were rarely influenced by the program because these were generally formed before using the program; rather, the program provided emotional reassurance to continue with current feeding plans.

Participants’ use of an mHealth program is critical if participants are to be exposed to the behavior change strategies underpinning the program’s effectiveness. Poor or limited engagement reduces the intervention “dose” received and limits the program’s effectiveness even if the behavior change strategies are sound. Our findings highlight the importance of understanding the unique needs of the intended users and how this might influence the mHealth design and delivery characteristics that are likely to be effective with those particular users. This fits with existing literature on user-centered design principles for developing mHealth programs [42].

Our finding that participants reported engagement with the program fluctuated according to need fits well with our quantitative analysis of predictors of actual app use based on analysis of app analytics [43]. In this analysis, first-time parents and those who registered when their infant was younger indeed had significantly higher levels of program use. These qualitative findings suggest that this was because of the higher learning needs of users at this time and their desire for quality information and support. This is in line with other effective face-to-face intervention programs targeting early-life obesity risk that have largely targeted first-time parents with young infants [44-46]. Understanding key infant feeding and developmental transition points (such as the introduction of solids) that may act as “sticky hooks” to engage parents in program content was also identified as important in this study. To achieve this, the program’s push notifications were specifically tailored to the infant’s age and stage of development and feeding method and pretested with parents to ensure the content and tone resonated with our target group [33]. Finally, understanding how the content may fit with predominant parenting beliefs and philosophies was identified as an important consideration. For example, our formative work suggested that some parents support reference to infant feeding guidelines in the program content, whereas others believed that guidelines were “too prescriptive” and approaches should be tailored to each individual baby [15].Understanding the genesis of beliefs and philosophies around infant feeding could potentially inform the tailoring of content. Clearly, getting the tone and balance of content “right” for the target group is important in maintaining engagement.

In line with previous research [26,30,47], our findings highlight the importance of mHealth design and delivery features in influencing engagement. For parents in our study, app mode of delivery was preferred over the website because of ease of access on their smartphone and the use of a combination of push notifications/text messages and emails was important for prompting program use. Again, this concurs with our quantitative analysis in which those using the app and receiving email notifications had higher levels of program use compared with those using the app alone [43]. This suggests that multiple points of contact with parents may promote better engagement. Consistent with previous research [48], the importance of having a credible content provider was a strong reoccurring theme in our findings and this was seen to enhance engagement with the program. This is not surprising given that although parents are increasingly relying on informal sources of support for infant feeding such as the Internet, family, and friends, they often report receiving conflicting information [49] and like the opportunity to cross-check with evidence-based recommendations. As expected, some technical glitches in the delivery of the program, including the temporary disabling of the app by new operating system updates, reportedly reduced engagement. This highlights the importance of extensive testing of the program across a range of devices before program launch and the need for ongoing app maintenance to accommodate operating system and other updates that might impact app functionality. For mHealth researchers, this will involve allowing time and budget for extensive beta testing and app maintenance during mHealth trials.

Participants reported that the program increased their confidence in feeding decisions by providing reassurance from a credible and trustworthy source, highlighting the interplay between program features and behavior change. Breastfeeding mothers reported that the program increased their confidence in their milk supply, which is critical given that a perceived lack of milk supply is the most common reason given for giving up breastfeeding in the literature [50]. Formula and mixed feeding mothers reported that the program gave them confidence to trust their infant’s hunger and fullness cues and not to pressure infants to finish all of the formula in the bottle. Given that responsive feeding in infants decreases the likelihood of rapid weight gain in infancy [5], this is an encouraging finding.

Our findings highlight the value of the Growing healthy program in providing an accessible “24/7” source of nonjudgmental support for infant feeding, potentially increasing participants opportunity for achieving optimal infant feeding practices. In particular, some mothers who mixed or formula fed felt that society provided little advice or support regarding how to use formula well. Consistent with our previous qualitative work [15], participants reported they often felt unsupported by health professionals in their decision to formula feed, with some viewing practitioners as “antiformula.” This is consistent with recent studies reporting that advice and guidance on formula feeding from health professionals is deficient and that parents typically rely on informal sources of support such as family, friends, and the Internet [51-53] to learn how to prepare and feed formula. Participants reported that the Growing healthy program helped to fill the void by providing a credible noncommercial source of information on formula feeding, and parents were receptive to messages about best practice formula feeding. The program also provided support when traditional sources of support such as health professionals were unavailable (eg, in the middle of the night) or difficult to access (eg, long wait times for a lactation consultant). Given that a trigger for behavior change can be situational and momentary [54] (eg, the urge to introduce formula to promote sleep in the middle of the night), having access to support at the exact time of need highlights one of the key advantages of mHealth programs over traditional face-to-face behavior change programs. Finally, participants reported that the program reinforced the advice received from health professionals and/or improved access to health professional support (particularly for breastfeeding), underscoring the potential value of mHealth programs in complementing health professional–delivered interventions to promote behavior change.

Our findings suggest that the program was less able to influence motivation in terms of infant feeding plans and intentions as these were reportedly formed before joining the program. It is likely that the timing of program delivery was an important limitation here. The average age of infants at the time of enrollment was 7 weeks, and around one-third of mothers had introduced formula at this time, limiting the ability of the program to influence plans around breastfeeding duration. Evidence suggests that plans about whether a mother will breastfeed and for how long are made antenatally, highlighting the importance of commencing the program before birth to influence goals, plans, and ultimately motivation for breastfeeding. Despite this, our findings suggest that mothers who were breastfeeding were motivated to continue because of the reassurance provided about their milk supply. Mixed feeding and formula feeding mothers were also motivated to practice responsive feeding by the reassurance that they were doing the “right thing.” This highlights the importance of reassurance as a motivator for continuation of desired infant feeding practices.

**Strengths and Limitations**

This study has a number of strengths and limitations. The use of qualitative methods is a strength in enabling an in-depth exploration of factors influencing engagement with the program, how the program influenced behavioral determinants, and the interplay between engagement and behavior change. However, social desirability bias is a potential issue, as some participants may have been eager to please researchers with positive accounts of the program and its effect and possible overreporting of desirable infant feeding practices. Nevertheless, this risk was minimized by having no contact between researchers and participants during the feasibility study and encouragement to provide their honest feedback to help improve the program. Furthermore, it is possible that those who volunteered to be interviewed were more engaged with and had more positive views about the program than those who declined or were unable to be contacted. However, there was no difference in the sociodemographic characteristics between those who participated and those who were uncontactable, suggesting no systematic bias.

### Conclusions

Our findings suggest that to maximize parental engagement, an mHealth program targeting infant feeding should come from an expert and credible source; be tailored to specific needs of the target group (eg, first-time mother, attitudes to parenting); use a combination of engagement strategies such as emails, push notifications, and text messages; and undergo extensive testing and ongoing maintenance to ensure high levels of functionality. Participants reported that the program enhanced confidence in breastfeeding and optimal formula feeding behaviors by providing a “24/7” accessible, expert, nonjudgmental support for infant feeding that complemented health professional advice. To improve the impact of the program on motivation and plans for breastfeeding, the program needs to commence antenatally and include behavior change strategies that specifically target motivation and intentions.

## Abbreviations

COM-B: Capability, Opportunity and Motivation, and Behavior.
mHealth: mobile health.
